# Dual Role of YY1 in HPV Life Cycle and Cervical Cancer Development

**DOI:** 10.3390/ijms23073453

**Published:** 2022-03-22

**Authors:** Alicja Warowicka, Justyna Broniarczyk, Martyna Węglewska, Wojciech Kwaśniewski, Anna Goździcka-Józefiak

**Affiliations:** 1 Department of Molecular Virology, Faculty of Biology, Institute of Experimental Biology, Adam Mickiewicz University, 61-614 Poznan, Poland; martyna.weglewska@amu.edu.pl (M.W.); agjozef@amu.edu.pl (A.G.-J.); 2 NanoBioMedical Centre, Adam Mickiewicz University, 61-614 Poznan, Poland; 3 Department of Gynecologic Oncology and Gynecology, Medical University of Lublin, 20-081 Lublin, Poland; wojciech.kwasniewski@umlub.pl

**Keywords:** YY1, transcription factors, HPV, cervical cancer, cell signaling, oncogenes, tumor suppressors, gene expression

## Abstract

Human papillomaviruses (HPVs) are considered to be key etiological agents responsible for the induction and development of cervical cancer. However, it has been suggested that HPV infection alone may not be sufficient to promote cervical carcinogenesis, and other unknown factors might be required to establish the disease. One of the suggested proteins whose deregulation has been linked with oncogenesis is transcription factor Yin Yang 1 (YY1). YY1 is a multifunctional protein that is involved not only in the regulation of gene transcription and protein modification, but can also control important cell signaling pathways, such as cell growth, development, differentiation, and apoptosis. Vital functions of YY1 also indicate that the protein could be involved in tumorigenesis. The overexpression of this protein has been observed in different tumors, and its level has been correlated with poor prognoses of many types of cancers. YY1 can also regulate the transcription of viral genes. It has been documented that YY1 can bind to the HPV long control region and regulate the expression of viral oncogenes E6 and E7; however, its role in the HPV life cycle and cervical cancer development is different. In this review, we explore the role of YY1 in regulating the expression of cellular and viral genes and subsequently investigate how these changes inadvertently contribute toward the development of cervical malignancy.

## 1. Introduction

Human papillomaviruses are small nonenveloped DNA viruses that cause nearly 5% of all human malignancies [1,2]. These pathogens are responsible for the development of six different cancers, such as cervical, vulvar, vaginal, anal, penile, and oropharyngeal [3]. Among these cancers, cervical cancer is the most clinically important, with more than 500,000 cases and 250,000 deaths annually [4]. While over 200 different HPV types have been identified, only a small subset is linked to the carcinogenic process. The most frequently occurring, cancer-causing, high-risk HPV types are HPV-16 and HPV-18. In contrast, low-risk HPV types, e.g., HPV-6 and HPV-11, do not cause cancers but are associated with benign epithelial condylomas [1,2,5].

All papillomaviruses have a double-stranded circular DNA genome of approximately 8 kb in length that is divided into three regions. The early region encodes six proteins (E1, E2, E4, E5, E6 and E7). E1 and E2 proteins regulate viral DNA replication and amplification, as well as viral gene transcription. E4, E5, E6 and E7 proteins play an essential role in the viral life cycle by creating an environment, within the differentiating epithelium, favorable for the replication of the viral genome and escaping immune response. The late region contains open reading frames for two viral structural proteins (L1 and L2) that form the viral capsid. The noncoding, long control region (LCR), also called as the upstream regulatory region, contains the binding sites for a number of transcription and regulatory factors that either activate or repress viral promoters, as well as for the viral E1 and E2 proteins that control viral replication and gene expression [1,2].

The HPV life cycle is closely linked to the epithelial differentiation of keratinocytes [2]. The virus enters the undifferentiated proliferating basal layers of the epithelium through microtraumas in the skin. As the cells differentiate, the viral genome is amplified, and new infectious virus particles are ultimately assembled and released from the upper terminally differentiated layers of the skin. Persistent infection with high-risk HPV types often leads to the integration of the HPV genome into the host chromosomes, thus causing uncontrolled expression of E6 and E7 viral oncoproteins and cervical cancer development [1,2,6].

The transcription of HPV is regulated during epithelial differentiation through different mechanisms, such as chromatin remodeling, histone modifications, DNA methylation, and binding of host transcription factors to LCR in the viral genome [7,8,9].

Yin Yang 1 is one of the transcription factors that bind to LCR and regulate the transcription of HPV viral oncogenes *E6* and *E7* [10,11]. Moreover, YY1 plays an essential role in carcinogenesis, as it controls the expression of various oncogenes and tumor suppressors, and regulates cell cycle, proliferation, differentiation, and apoptosis [12]. It is worth mentioning that altered expression of YY1, which regulates both viral and cellular mechanisms, may result in virus-related carcinogenesis and can be considered to be a common characteristic of these types of tumors.

In this review, we described the complex role of the YY1 protein in the viral life cycle and cancer development, particularly emphasizing the role of YY1 in the HPV life cycle and cervical cancer development.

## 2. Structure and Function of YY1

YY1 is a multifunctional transcription factor with an indispensable role in the proper functioning of eukaryotic cells by influencing various biological processes. YY1 belongs to the Krüppel-like family of transcription factors that are described as zinc-finger proteins with dual activity, that is, they act either as positive or negative regulators. This protein was independently discovered and named in 1991 by Shi et al. as YY1 [13], by Park and Atchinson as nuclear factor E1 (NF-E1) [14], and by Hariharan et al. as delta (δ) [15]. Later, human YY1 was further referred to as upstream conserved region binding protein, nuclear matrix protein 1, nuclear factor D (NF-D), or F-ACT1 [16].

**Figure 1:**
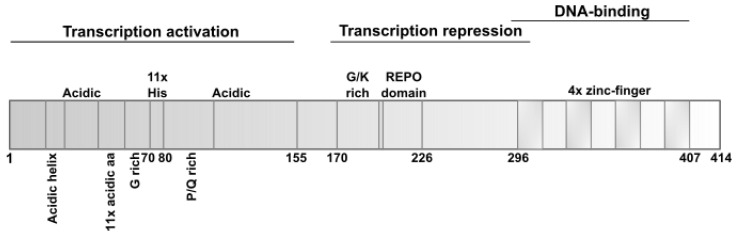
Schematic representation of the YY1 domains. A transactivation domain was recognized at the N terminus (1–155 aa) of YY1 protein. This activity is mediated by the acidic amino acid residues of the amphipathic, negatively charged helix (16–29 aa), 11 consecutive acidic residues (43–53 aa), glycine-rich motif (G-rich, 54–69 aa), and proline/glycine-rich motif (P/Q-rich, 80–100 aa). Between them, there is a histidine tract with 11 histidine residues (70–80 aa) responsible for nuclear accumulation and YY1-mediated RNA metabolism. In the central sequence, a slightly overlapping C terminus is located in the transcription repression domain. This domain includes the glycine/alanine-rich region (G/A-rich, 154–170 aa) that mediates DNA interaction and, together with glycine/lysine-rich region (GK-rich, 170–200 aa), plays role in YY1-dependent acetylation/deacetylation events of the REPO domain (205–226 aa) that recruits corepressors of the polycomb group protein. The C terminus consists of four zinc-finger motifs (296–320 aa, 325–347 aa, 353–377 aa, and 383–407 aa) which guarantee YY1 interaction with DNA.

The human *YY1* gene is located on the telomeric region of chromosome 14 (14q32.2) and encodes 414 amino acids. The gene produces eight putative proteins (a, b, c, d, e, f, g, and h) that are generated by an alternative splicing mechanism, and their functions are still unknown. The molecular weight of YY1 has been estimated to be 68 kDa (predicted weight being 44 kDa), and the protein contains multiple domains (Figure 1) [16]. The N terminus of YY1 (1–155 aa) acts as a transactivation domain and is involved in the electrostatic interaction with positively charged proteins, owing to the acidic nature of some residues [12]. The histidine tract (contains 11 histidine residues) within the N-region is responsible for the nuclear localization of YY1. The repressive capacity of YY1 is controlled by the central region (170–226 aa) and glycine/lysine-rich sequence (333–397), which overlap zinc fingers near the C terminus. In the C-terminal region (296–407 aa) of YY1, four C_2_H_2_ zinc-finger motifs have been recognized which mediate the binding of a protein molecule to nucleic acid [17,18,19].

YY1 is highly expressed in a variety of mammalian tissues and evolutionary conserved across species [16]. The YY1 promoter does not contain the classic TATA box region but contains Sp1-binding sites and is rich in CG dinucleotides, which mimics promoters of important regulators and housekeeping genes. This characteristic feature suggests the importance of YY1 in crucial biological mechanisms such as development [20].

The YY1 protein can form homodimers and bind both DNA, via its four zinc fingers, and single-strand RNA, via zinc fingers and/or the N-terminal region [21,22,23]. The DNA-binding motif of YY1 is 9–11 nucleotides long, and the consensus sequence is represented by 5′-CGCCATnTT-3′. YY1 can recognize a longer sequence (5′-GGCGCCATnTT-3′ or 5′-CCGCCATnTT-3′) with the 5′-GCCAT-3′ core motif [24]. Most of the YY1-binding sites are close to the transcription initiation sites, or sometimes they are present distal to the regulated genes [25]. RNA binding is considered to be less specific, and to date, the complex formed between YY1 and long noncoding Xist RNA is the best recognized complex [23,26]. In this case, YY1 binds both DNA and RNA, mediates X-chromosome inactivation by docking Xist onto the X-chromosome, and also regulates Xist expression [26,27]. Recent studies also indicate that YY1 interacts with many other microRNAs (miRNAs) and thus influences different biological pathways, for example, miR-1, miR-133, and miR-34c during myogenesis [28,29].

YY1 plays an important role in many essential biological processes, such as transcription regulation, control of protein activity, and cell cycle progression, as well as for survival, proliferation, and differentiation of many different cell types. YY1 controls the expression and interacts with a large subset of genes involved in basic metabolic pathways, protein synthesis, and, surprisingly, some viral genomic elements at different stages of infection.

A characteristic feature of YY1 is its bifunctional nature, that is, it acts both as a transcription activator and repressor. This dual nature of YY1 was originally observed during the conversion of YY1 from a repressor to an E1A [30]. The ability to control expression is modulated by different mechanisms, either direct or indirect, depending on the interacting protein, regulated promoter, and chromatin structure [16]. On the other hand, the biological functions of YY1 are regulated by its post-translational modifications, such as phosphorylation, SUMOylation, ubiquitination, acetylation, or methylation, but also depend on the accessibility and concentration of specific enzymes or protein complexes [18,31,32,33].

The transcription activity of YY1 could be regulated by either acetylation or deacetylation. The YY1 protein can be acetylated by p300 or PCAF at two regions [31]. The modified central, glycine/lysine-rich motif guarantees repressive activity and could be deacetylated by HDAC deacetylases, while the acetylation of C-end zinc fingers is stable and reduces the ability of YY1 to bind DNA. Furthermore, it is also assumed that YY1 indirectly regulates the expression of genes by targeting acetyltransferases or deacetylases to specific locations which mediate rearrangement of the histone landscape and transcriptional alterations [31].

In addition to acetylation, YY1 can recruit other chromatin modification complexes, such as methyltransferase PRMT1 and nucleosome remodeling complexes INO80 or BAF, that positively regulate transcription [34,35,36].

YY1 acts as an activator or repressor depending on the cellular context. YY1 binds corepressors or coactivators and directs them directly to a promoter sequence for boosting or halting transcription. Previous studies suggested the presence of interacting proteins [12,16,32], such as proteins of the basic transcription machinery (RNA polymerase II, TIIB, TATA-binding protein, etc.), DNA-binding activators (SP-1, c-MYC, NF2, CREB, ATF6, etc.), and transcription modulators (CBP, HDACs, p300, PARP1, and DMNTs). In addition, YY1 can regulate transcription indirectly by physically blocking the DNA region from binding to other proteins [19].

It is worth mentioning that vertebrate YY1 was identified to be a homolog of the Drosophila Pleiohomeotic sequence, and both belong to the polycomb group proteins (PcGs). The short (25 aa) REPO domain of YY1 interacts with a protein complex consisting of other PcGs, and the role of YY1 is to target the whole complex to the DNA sequence. This interaction stabilizes transcription repression by epigenetic histone modification, which usually involves methylation of lysine 9 and 27 residues on histone H3 and/or deacetylation of lysine 9 and 14 residues on histone H3 [37]. The regulation of embryonic development, cell proliferation and differentiation, hematopoiesis, B-cell development, and other biochemical mechanisms that are regulated by PcGs–YY1 activity mainly depend on the above-mentioned mechanism [38,39].

It is noteworthy that the interaction between YY1 and PcG proteins mediates methylation of both histones and DNA and leads to gene silencing. Regulation of DNA methylation depends on the recruitment of DNA methyltransferases (DNMTs) to the repressed DNA sequence [40]. The role of YY1 in regulating DNA methylation has undoubtedly been proven in various cancers. The YY1/PcG/DNMT complex via changes in methylation status is important for the development of hepatocellular carcinoma and cervical cancer as well as leukemias such as chronic myeloid leukemia or acute myeloid leukemia [12,41]. For example, YY1 is involved in the repression of the tumor suppressor CEBPD by mediating interaction between polycomb subunit SUZ12, DNMTs and the *CEBPD* gene promoter [42]. Moreover, overexpressed YY1 is involved in cell proliferation, migration and invasion in highly aggressive triple-negative breast cancer (TNBC). Recently, Shien et al. showed that in case of TNBC overexpressed YY1 upregulate expression of long noncoding RNA (lncRNA) Kcnq1ot1 which interacts with DMNT1 and causes methylation and decreased expression of the *PTEN* tumor suppression gene [43]. 

Importantly, YY1 does not always function as a classic transcription factor. To regulate mammalian gene expression, YY1 could also act as a structural protein that controls transcription by facilitating enhancer–promoter interactions [44]. This architectural protein binds to hypomethylated DNA regions either at enhancer or promoter-proximal elements and then dimerizes, induces DNA looping, and finally mediates the interaction between these sequences. The YY1-mediated association between regulatory elements activates or represses gene transcription depending on the nature of these sequences, but it should be noted that this interaction plays a crucial role in tumorigenesis. The importance of this mechanism is demonstrated in experiments where deletion or depletion of YY1 completely disrupts DNA looping and alters gene expression [44,45]. It has recently been reported that the DNA looping mediated by YY1 and CCCTC-binding factor (CTCF) alters the chromatin architecture and transcription regulation in neural progenitor cells [46].

Researchers estimate that YY1 may be involved in the transcription of approximately 7–10% of mammalian genes [47,48]. This transcription factor activates, represses, or induces the expression of a broad range of promotors and genes [16,48,49] that are essential for development, cell signaling (e.g., control of p53 level), cell cycle progression (e.g., phosphorylated YY1 reactivates genes that are the indicators of interphase entry, regulates G2/M transition, and by binding to Rb induces cell-cycle arrest), antiviral response (e.g., modulation of interferon production), and cell homeostasis and stability of DNA (e.g., regulation of PARP-1 expression) [50,51,52,53,54,55].

YY1 can potentially activate various pluripotent genes (e.g., *Nanog, Oct4,* and *Myc*) and hence is an essential factor for embryonic viability, vertebrate development, and organogenesis [56,57]. YY1 was reported to be an important factor for trophoblast invasion and migration at the early stage of pregnancy, and the decreased level of this protein can be used as a diagnostic biomarker for miscarriage [58]. In addition, YY1-deficient mouse embryos are defective in this protein and do not survive after a short period of implantation, which can be attributed to the dysregulated expression of early proliferation and/or differentiation genes. On the other hand, YY1 heterozygotes (with one mutated allele) are developmentally retarded and show neurulation defects such as asymmetric brain structure [59]. These observations established that YY1 also plays a pivotal role in the development and functioning of the central nervous system [20]. In humans, deletion or missense mutation of YY1 does not cause lethal effects but leads to the development of Gabriele-de Vries syndrome (also known as YY1 syndrome), which is characterized by mental retardation and other neurological abnormalities [25,60,61]. YY1 is one of the principal factors that are involved in the neurodevelopment process by controlling epigenetic status (mainly H3K27 methylation) and transcription of early genes and regulators, such as *Wnt1* and *p53* [25,61]. In addition to early genes, YY1 directly targets other genes crucial for normal brain functions [20]. In addition, YY1 is also a factor that determines proliferation and differentiation of neural progenitor cells through associations with lncRNA Sox2ot and other types of neural cells, such as neurons or astrocytes [20,56,62].

YY1 also exhibits a protective role in the nervous tissue. Together with NRF2, the protein regulates neuronal signaling pathways and enhances the transcription of antioxidant enzymes that protect individuals against ischemic stroke or Parkinson’s disease [63,64].

However, the role of YY1 is not restricted to neurogenesis during organogenesis. YY1 is also involved in, for example, skeletal muscle differentiation. Together with the PcG EZH2, multifunctional transcription factor NF-κB, and HDAC1, YY1 acts as a negative regulator of late-stage differentiation genes during myogenesis [57,65,66].

Previous studies have also shown that YY1 is a critical regulator of B-cell progenitors at all stages of differentiation, and deletion of the *YY1* gene completely disrupts this process. Additionally, YY1 plays a role in the V(D)J gene rearrangement, which is important for the development of adaptive immune response [67,68]. In B lymphocytes, YY1 binds to promoters of important genes involved in mitochondrial bioenergetics, ribosomal function, or splicing mechanisms that regulate key pathways involved in the development of this subset of blood cells [69].

## 3. The Dual Role of YY1 in HPV Life Cycle and Cervical Cancer Development

### 3.1. YY1 Protein Controls the Expression of Viral Oncogenes E6 and E7 during HPV Productive Life Cycle

HPVs are known to infect stratified epithelium, and their productive life cycle is strongly associated with the differentiation program of the epithelial cells [2]. The complex life cycle of HPV initiates in undifferentiated basal epithelial cells where the expression of *E6* and *E7* genes is restricted, most likely to limit host immune response and because their role in the cell cycle to maintain the expression of the cellular DNA replication machinery is less important in actively dividing epithelial cells. After the differentiation of epithelial cells, the production of early transcripts of the virus is increased, resulting in higher E6/E7 protein expression and activation of the late promoter, which further enhances the expression of late virus genes in the upper layers of the squamous epithelium. The elevated expression of E6 and E7 proteins is important to maintain the proliferation of host cells and to provide viral access to the host cell DNA replication machinery. Moreover, it has been shown that amplification of the viral genome in the differentiated epithelial cells requires extensive expression of the E6 protein [1,2,6].

The expression of HPV oncogenes during the viral life cycle is regulated by the early (E) promoters (PE: P97 in HPV-16 and P105 in HPV-18), while the expression of late viral genes is controlled by late (L) promoters (PL: P670 in HPV-16 and P811 in HPV-18). The switch between HPV promoters and the HPV gene expression might also be modulated by the binding of cellular transcription factors, which are differentially expressed in various epithelial layers, to the noncoding LCR region in the HPV genome [7].

The noncoding LCR is constituted by approximately 850 bp (7–11% of the total genome) and is located between the end of L1 and the beginning of E6 sequences. Four E2-binding sites divide LCR into three functionally distinct segments: 5’ (distal), central, and 3’ (proximal) regions. The 5’ segment of LCR is about 300 bp in size and contains transcription termination and polyadenylation sites for late transcripts and regulatory elements that control late mRNA stability. The central segment is about 400 bp long and functions as a transcription enhancer. The 3’ segment is approximately 140 bp in length and contains the E2-binding site, which plays a role in initiating viral replication and may also be involved in the transcriptional regulation of the *E6* and *E7* genes [64].

**Figure 2:**
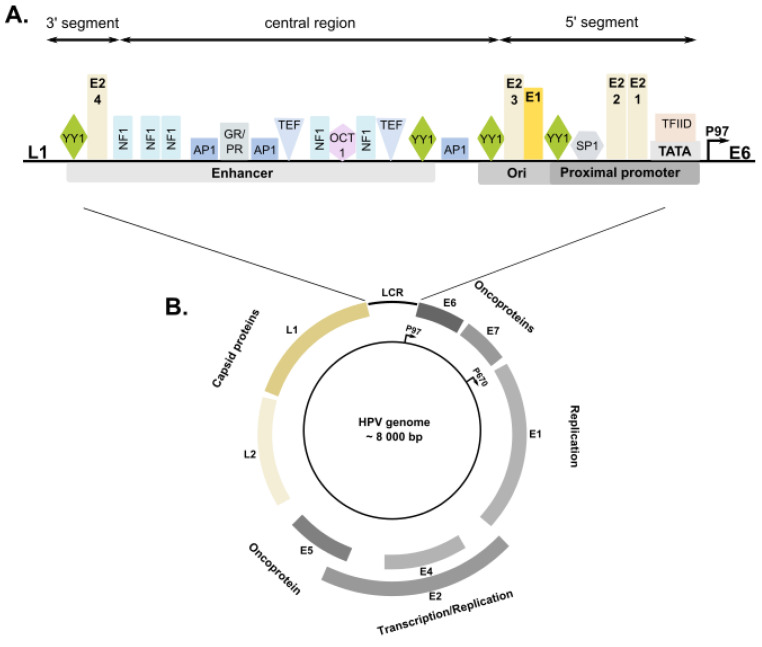
The organization of HPV genome and LCR (long control region) sequence. (**B**) Schematic map of HPV genome. All papillomaviruses have a genome of approximately 8 kb in length, which is divided into three regions. The early region encodes six proteins (E1, E2, E4, E5, E6, and E7), and the late region codes for two structural proteins (L1 and L2). The noncoding LCR contains the major cis-regulatory sequence, which is involved in the initiation and regulation of replication. In addition to this, it controls the expression of *E6* and *E7* oncogenes. The early (P97) and late (P670) promoters are marked by arrows. (**A**) Representative structure of LCR (region of control of early promoter P97). The fragment of the LCR sequence (central region and 3’ segment) was enlarged to allow visualization of the E1- and E2-binding sites, the TATA element of the P97 promoter, and the binding sites of most important transcription factors, such as AP-1, GR/PR (glucocorticoid and progesterone receptor), NF-1, SP1, OCT-1, TEF-1, TFIID, and YY1. Note that only a limited number of binding sites are illustrated. There are many known binding sites, but it is not possible to illustrate all the transcription factors and their precise binding sites.

LCR contains binding sites for a plethora of transcription factors that either activate or repress the E and L promoters, such as AP-1 (activator protein 1), NF-1 (nuclear factor 1), SP-1 (specificity protein 1), OCT-1 (octamer-binding protein 1), TEF-1 (transcription elongation factor 1), TFIID (transcription factor II D), and YY1 [70,71] (Figure 2).

The above-mentioned facts clearly indicate that YY1 is a multifunctional transcription factor that can repress or activate transcription, depending on the promoter sequence and physiological context. Several potential YY1-binding sites within the LCR of HPV-16 enhancers have been identified. However, only the cluster of five sites located close to the AP-1-binding site was found to be responsible for the repression of the HPV-16 P97 promoter. It was shown that the binding of YY1 to this region exerts strong repressive effects on E6 and E7 transcription by masking the AP-1-binding sites [10,72]. It has also been demonstrated that YY1 and TEF-1 function cooperatively, that is, by binding and activating the 5’ distal enhancer within the LCR of HPV-31 [73]. Moreover, it was proved that the binding of YY1 to this site increases as cells differentiate [74]. Interestingly, the binding of YY1 to the 5’ distal enhancer shows minimal effects on the transcription of HPV-16 early genes [72].

**Figure 3:**
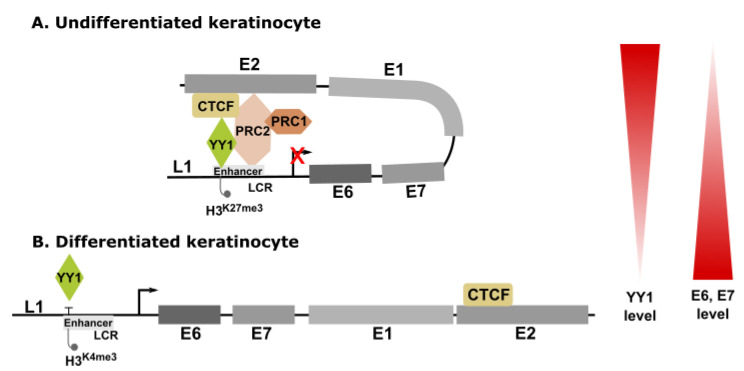
Model showing the role of YY1 during HPV life cycle in undifferentiated (A) and differentiated keratinocytes (B). The life cycle of HPVs is strongly associated with the differentiation program of the epithelial cells. HPV infection initiates in the undifferentiated basal epithelial cells where the expression of E6 and E7 genes is restricted by the establishment of a CTCF (CCCTC-binding factor)–YY1-dependent chromatin loop of epigenetically repressed chromatin (A). Upon epithelial differentiation, the production of early transcripts of virus is increased. In differentiated keratinocytes, the elevated E6 and E7 protein levels are important to maintain host cell proliferation and provide viral access to the host cell DNA replication machinery. This is synchronized with a differentiation-induced reduction in YY1 levels, which releases the repressive chromatin loop and attenuates the recruitment of polycomb repressor complexes (PRC1 and PRC2) and inhibits H3K27me3 (trimethylation of histone H3 at lysine 27) deposition. Increased accessibility and reduced H3K27Me3 at the viral enhancer sites result in derepression of the early promoter, increased RNA Pol II recruitment, and H3K4me3 (trimethylation of histone H3 at lysine 4) deposition, resulting in increased E6/E7 oncogene expression.

HPV genomes are wrapped around histone proteins to form nucleosomes, which package together to produce chromatin [75,76]. Thus, it is clear that the HPV gene expression during the viral life cycle in different layers of epithelium might also be regulated through epigenetic mechanisms such as post-translational modifications of histones, including acetylation, phosphorylation, and methylation, as well as methylation of DNA [8,9]. Interestingly, it was found that YY1 can regulate the transcription of early genes of HPV during the differentiation-dependent viral life cycle through histone modifications. YY1 facilitates the recruitment of the polycomb repressor complexes 1 and 2 (PRC1 and PRC2) to the LCR region in viral chromatin [77,78,79] (Figure 3). PRC1 catalyzes H2AK199Ub deposition, while PRC2 catalyzes H3K27Me3 deposition, which leads to the repression of early promoter activity and expression of viral oncogenes [79]. Further studies showed that YY1 associates with chromatin organizing CCCTC-binding factor and is involved in the formation of the LCR-E2 ORF (open reading frame) chromatin loop. Thus, the complex between YY1 and the transcription regulator CTCF coordinates the expression of oncogenes by facilitating the HPV-18 DNA loop formation, recruiting the polycomb complex, and mediating epigenetic modification [79]. The CTCF protein binds to the DNA molecule and plays a fundamental role in the three-dimensional organization of chromatin. High-risk HPV types contain a conserved CTCF-binding site in the E2 ORF region. It has been proven that mutation in this particular site due to the integration of the HPV-18 genome and CTCF depletion results in an increased level of E6/E7 transcripts [80]. Moreover, it was demonstrated that the reduced expression of YY1 upon differentiation disrupts the LCR-E2 ORF chromatin loop, leading to enhanced E6/E7 expression that further supports the productive viral life cycle [79]. All these studies confirmed that YY1 might also regulate the levels of E6 and E7 transcripts through epigenetic mechanisms (Figure 3).

### 3.2. YY1 and Cervical Cancer

E6 and E7 oncoproteins also play a crucial role in HPV-associated carcinogenesis. However, it has been suggested that HPV infection alone may not be sufficient to induce and promote cervical cancer. Indeed, a number of additional factors whose dysregulation drive the oncogenic process, which occurs in concert with high-risk HPVs E6 and E7 oncoproteins, are required to establish the disease [81,82] Moreover, alternations in mitochondrial DNA are associated with cervical cancer development [83].

Both E6 and E7 proteins continue to be expressed in tumor-derived cells and are required for the maintenance of the transformed phenotype. Loss of expression of either E6 or E7 proteins induces cell growth arrest and apoptosis. One of the most important functions of E6 and E7 in HPV-dependent carcinogenesis is their ability to inactivate the p53 and pRb (retinoblastoma) pathways, which subsequently disrupt the control of cell regulatory pathways such as apoptosis and cell cycle. Decades of research on the HPVs provided a better understanding of the role of E6 and E7 proteins in the development of cervical cancer. Now, it is well known that the functions of HPV oncogenes can be linked with all “characteristic features of cancer”, such as sustaining proliferative signaling; evading growth suppressors; enabling replicative immortality; activating invasion and metastasis; enabling replicative immortality, genome instability, and mutation; inducing angiogenesis; resisting cell death; and deregulating cellular energetics [84,85,86,87,88].

The expression of E6 and E7 oncoproteins in cancer cells might be regulated through several different mechanisms, and the role of YY1 in this process has not been clearly defined yet. It is also unclear if the mechanism of differentiation-dependent regulation of HPV oncogene expression plays a role in HPV-driven cancers.

In most cases (70–85%), uncontrolled E6 and E7 expression is the result of the integration of the genome of high-risk HPV into the host chromosome. During the integration process, the early P97 promoter and E6 and E7 coding sequences are maintained, whereas other regions of the viral genome are deleted, or their expression is disturbed. The integration leads to the loss of expression of the viral E2 transcriptional repressor, which can no longer control the P97 promoter to restrict E6 and E7 expression. Approximately 15% of cases of cervical cancers contain episomal forms of HPV genomes. In these conditions, E6 and E7 expression is increased despite the presence of the E2 protein, possibly through epigenetic and/or chromatin conformation changes in the viral genome close to the P97 promoter [6].

YY1-binding sites in the LCR region of the HPV-16 genome are often mutated in cervical cancer cells, and it was suggested that mutations affecting YY1 motifs might be one of the mechanisms that enhance viral oncogene expression during the cancer progression [89,90,91,92,93].

On the other hand, the negative control of viral transcription was observed when YY1 binds the promoter elements of the E6–E7 promoter that regulates the expression of oncoproteins during the life cycle of HPV-16 and HPV-18 [72,94,95].

However, further studies are required to elucidate the role of YY1 in the regulation of E6 and E7 expression during HPV-induced carcinogenesis.

Apart from controlling the expression of HPV oncogenes E6 and E7, YY1 is also involved in the regulation of transcriptional activation and repression of many genes associated with multiple cellular processes, and disturbances in these mechanisms can lead to the development of cervical cancer.

The expression of YY1 is found to be increased in HPV-16- and HPV-18-positive cervical cancer cells [96]. It has also been shown that the depletion of YY1 levels in HPV-18-positive HeLa cells results in increased p53 expression and apoptosis. Further studies demonstrated that elevated YY1 levels contributed to drug resistance in cervical cancer patients. Specifically, treatment of HeLa cells with arsenic trioxide was shown to reduce YY1 expression, which was correlated with increased apoptosis, suggesting that YY1 could be a potential anticancer target for the treatment of cervical carcinoma [97]. In addition, it was shown that the expression of YY1 in cervical cancers cells is positively correlated with the E6 protein of HPV-16 and negatively correlated with E-cadherin expression [36]. E-cadherin is critical for the formation and maintenance of adherent junctions in areas of epithelial cell–cell contact, and loss of this protein increased the invasiveness and metastasis of cervical cancer cells and other tumors [98].

Recent studies have shown that epigenetic modifications, including deregulation of miRNA, lncRNA, and circular RNA levels, also play important roles in cell transformation during different stages of cervical cancer development [99]. Interestingly, it has been shown that microRNA-181 (miR-181) targets YY1 expression and inhibits cervical cancer progression, while overexpression of miR-181 inhibits cervical cancer cell growth in vitro and in vivo by regulating YY1 expression [100]. Moreover, it was demonstrated that YY1 and HPV E7 promote the expression of the lncRNA FANCI-2, thus contributing to cervical cancer development [101].

All the above-mentioned findings suggest that besides regulating the expression of viral oncogenes E6 and E7, YY1 might also be involved in the control of other processes crucial for the progression of cervical cancer. Further studies are required to identify the signaling pathways that are regulated by YY1 and to understand the mechanism underlying the action of YY1 during cervical cancer development.

## 4. YY1 and Its Contribution to other Cancers

Several studies have been increasingly conducted worldwide to determine the oncogenic role of YY1 in different cancers other than cervical cancer. YY1 mediates the expression of many genes involved in cell proliferation, differentiation, and apoptosis, and hence its role in cancer progression has been a topic of interest among researchers [48]. Depending on the cellular conditions and protein interactions, YY1 may act as a stimulator or suppressor of tumor growth. Activation of YY1 stimulates uncontrolled cell proliferation, induces resistance to apoptosis, enhances angiogenesis, destabilizes the genome, and causes accumulation of mutations that can ultimately lead to the enhanced potential for tumorigenesis and metastasis. The affected pathways mainly include downregulation of p53 activity; interference with poly-ADP-ribose polymerase activity; changes in the expression of c-Myc, NF-κB, and other transcription factors; and induction of production and secretion of inflammatory mediators (such as interferons) [12,16,48]. YY1 overexpression has previously been observed in different types of cancers, such as breast, prostate, ovarian, colon, melanoma, and acute myeloid leukemia [12,48]. In some of these cancers, increased YY1 level is associated with poor prognosis (e.g., prostate and breast), while in others, it induces mechanisms that inhibit tumor progression (e.g., ovarian and colon) [102]. Unfortunately, the underlying mechanisms are not clearly understood, but they are undoubtedly related to the YY1 interaction network and YY1-dependent regulation of genes and/or expression of noncoding RNAs (miRNA and lncRNA) [12,47]. Recently, research concerning the role of YY1 in the development of squamous cell carcinoma (SCC) gained greater attention. In some cancers, certain HPV strains/types are considered to be etiological risk factors. YY1 overexpression was established in oral and oropharyngeal squamous cell carcinoma (OSCC) [103]. Additionally, it has been shown that YY1 is a positive regulator of the coactivator-associated arginine methyltransferase 1 (CARM1) gene promoter. The association of YY1 with CARM1 promotes carcinogenesis in oral tissues [103]. Previous studies established that overexpression of CARM1 is associated not only with oral cancer, but also with other cancer types such as breast cancer, colorectal cancer, and prostate cancer.

Furthermore, YY1 is overexpressed in esophageal squamous cell carcinoma (ESCC), where overexpression enhanced the progression of ESCC and correlated with a negative prognosis. Moreover, high expression of YY1 was detected in laryngeal cancer [104] and head and neck squamous cell carcinoma (HNSCC), including nasopharyngeal cancer (NPC) [105]. Recent studies have established that HPV infection is a well-known risk factor for HNSCC [106] and oropharyngeal cancer [107]. Overall, YY1 is known as a negative regulator of HPV-16 E6 and E7 oncoprotein expression during the HPV life cycle, and a high level of YY1 expression prevents the transcriptional activity of HPV in the infected tissue. The analysis of the DNA sequence obtained from HPV-16 in HNSCC cases showed the deletion of YY1-binding sites in the viral LCR region [108]. YY1 plays a regulatory role for various host factors/host protein partners and is tightly associated with cancerogenesis. In NPC, YY1 mediates repression of TRIM26 [109] and suppression of tumor growth by inactivating the transcriptional activity of c-Myc. Thus, YY1 inhibits the expression of noncoding RNA miR-141, which is mediated by c-Myc [110]. Moreover, overexpression of YY1 inhibits NPC cancer cell proliferation and promotes apoptosis. YY1 can suppress apoptosis via inhibiting MYCT1 in laryngeal cancer cells. YY1 binds to the MYCT1 promoter region and blocks its transcriptional activity [104]. Analysis of the mRNA level of *YY1* showed that *YY1* mRNA expression is associated with the mRNA expression of the transcription factor *LSF* [111]. Furthermore, co-expression of these two transcription factors, YY1 and LSF, was recently detected in hepatocellular cancers [112]. Interestingly, it has been observed that YY1 plays a role in the development of resistance to chemotherapeutic agents. Cisplatin, the first-line drug for the chemotherapy of many cancers, including NSCC, was found to upregulate YY1 expression. Overexpression of YY1 upregulates phosphorylation of protein kinase B (AKT), which is responsible for chemoresistance to anticancer drugs. Knockdown of YY1 sensitized NSCC cells to cisplatin and enhanced the anticancer effect of the drug [113]. Knockdown of YY1 mediates inhibition of cell proliferation, migration, and development of NSCC. On the other hand, some observations indicate that higher levels of YY1 facilitate anticancer drug response, and in many cases, knockdown of the *YY1* gene results in a better prognosis to treatment [113,114,115]. YY1 appears to be a potential drug target and a diagnostic or prognostic marker for the detection of cancers [12,32,115].

The regulating role of YY1 in various cancers is summarized in Table 1.

## 5. YY1—Activator or Repressor of Viral Expression?

In addition to the ability to modulate gene transcription in HPV, YY1 can repress or activate gene transcription in several viruses, including human immunodeficiency virus (HIV-1), adenovirus, adeno-associated virus (AAV), parvovirus, Moloney murine leukemia virus (M-MLV), and herpesviruses [117]. The initial observations indicate that YY1 functions as a transcription initiator in the complex having the P5 promoter of AAV and acts as a bifunctional protein on interaction with the AAV E1A protein [30,118]. Activation of viral expression was also reported for retroviruses, including human T lymphotropic virus type 1 (HTLV-1) and HIV type 1 (HIV-1). YY1 acts as a potent transactivator of *HTLV-1* gene expression. In contrast to HIV-1, YY1-binding sites/responsive elements are located in the HTLV-1 R region. After the interaction between YY1 and HTLV-1 RNA, the rate of initiation and elongation of viral transcription was significantly increased [119].

The YY1 protein can perform dual functions. YY1 is involved in the elevation of HIV-1 replication and also exerts a positive effect on the production of the HIV-1 virus. YY1 can act as a transcriptional activator through upregulation of the activity of U3 and U3RU5 promoters. YY1 overexpression leads to an increase in viral mRNA levels [120]. Interestingly, it has been observed that YY1 can act not only as a transcriptional activator in HIV-1 gene expression, but also can bind to the HIV-1 long terminal repeat region and repress viral expression [121]. Moreover, overexpression of YY1 promotes HIV-1 repression and facilitates the establishment of latency [122]. Along with host transcription factor LSF, YY1 recruits HDAC1 deacetylase to the viral LTR and attenuates HIV-1 transcription [120,121,123].

Furthermore, it has been demonstrated that YY1 physically interacts with retroviral integrases. A previous study reported that YY1 binds to the integrases of HIV-1, M-MLV, and avian sarcoma virus, which consequently increases the activity of these enzymes and facilitates integration events. Studies revealed that this interaction facilitates the integration of M-MLV cDNA into the host chromosomes [124]. Moreover, YY1 interacts with Trim28, an M-MLV protein that regulates viral gene expression. YY1 and Trim28 form a silencing complex and mediate the silencing of the M-MLV protein in embryonic cells, thus restricting viral replication. It is worth noting that the YY1–Trim28 complex is mainly detected in the embryonic cells [125].

Recently, the role of YY1 in establishing coronavirus infection was also noticed, but the underlying molecular mechanism still remains unknown. A recent study by Morenikeji et al. [126] established that YY1 targets seven genes (*TLR7*, *TLR9*, *CEBPD*, *CEBPD*, *IRF1*, *IL-6*, and *RHOA*) that are significantly expressed during bovine coronavirus infection. Many of these genes are implicated in the development of antiviral host immune response and proinflammatory pathways. YY1 has the capacity to either regulate or alter (activate or repress) the expression pattern of identified genes, and hence this protein acts as a potential target in anti-BCoV therapy.

YY1 is also known to interact and regulate the life cycle of another RNA virus, that is, hepatitis delta virus (HDV). It has been demonstrated that two isoforms of the HDV antigen (HDAg) interact directly with YY1 and together with acetyltransferase p300/CBP form a large nuclear complex that enhances viral replication [127]. YY1 also plays an important role in the life cycle of the hepatitis B virus (HBV). HBV is the major cause of hepatocellular carcinoma, but the mechanism of pathogenesis is still unknown and not well understood. It is a well-known fact that viral infection alters the expression of cellular miRNAs. Recent data demonstrated that HBV suppresses DGCR8 promoter activity and thus disrupts the processing of miRNAs. Although the exact mechanism is still unknown, detailed analysis showed that YY1 is involved in this mechanism. Recent studies showed a different kind of association between YY1 and HBV infection. A previous study demonstrated that during HBV infection, the rate of YY1 expression increased significantly, which indicates that HBV activates YY1 expression [128]. Previous data revealed that HBV induces YY1 expression through the MAPK signaling pathway [128]. It has been shown that YY1 directly inhibits the activity of the DGCR8 promoter [129], suggesting that YY1 regulates miRNA expression. In addition, the HBx viral protein could enhance YY1 activity through the recruitment of HDAC [130]. It has been proven that HBx can interact with YY1 [131]. Other data revealed that YY1 can bind to the binding elements of the Sox4 promoter, thus stimulating its activity during HBV infection. Moreover, studies showed that YY1 downregulates miR-335, miR-129-2, and miR-203, and subsequently, HBV post-transcriptionally upregulates Sox4 expression [128]. The YY1-mediated positive regulation of viral transcription has been also reported for the HLJ1 (heat shock protein) of HBV, especially at an early stage of infection [32,120,132,133]. Taken together, HBV can regulate Sox4 expression by two independent pathways for which YY1 is required.

During viral infection, the HBV genome exists in the nuclei of infected hepatocytes as a minichromosome (covalently closed circular (cccDNA)). The YY1 protein can bind to cccDNA of HBV and regulate the expression of viral genes. Furthermore, YY1 mediated the specific interaction between HBV cccDNA and the 19p13.11 region of the cellular chromosome. This chromosome region contains an enhancer sequence, which is modulated by YY1 (the binding elements of YY1 are located in 19p13.11 enhancer site). Suppression of YY1 expression resulted in decreased transcriptional activity of HBV cccDNA. In conclusion, the fundamental role of YY1 in HBV infection suggests that YY1 is a novel target for anti-HBV therapy.

It has been shown that YY1 regulates the expression of herpesvirus genes. Several reports have demonstrated the presence of binding sites for cellular transcription factors, including YY1, within the herpesvirus genomes. To date, the exact mechanism behind the maintenance of viral latency and viral reactivation to a lytic state is still unknown. It is well known that a number of host factors are involved in the establishment of a persistent viral infection. Studies have shown that one of the factors, that is, cellular YY1, plays an important role in the maintenance of herpesviral latency. Liu et al. showed that YY1 is important for the establishment of human cytomegalovirus (HCMV) latency [134]. The exact mechanism has been proposed by Poole et al. [135]. The authors demonstrated that bone morphogenetic protein receptor type 2 plays a crucial role in the latent infection of HCMV by suppressing the tumor growth factor-beta (TGFbeta) signaling pathway. Suppression of TGFbeta signaling leads to the downregulation of the expression of the cellular miRNA (hsa-miR-29a) and then to the stabilization of YY1 and repression of the MIEP viral promoter. As a result, the latent HCMV infection is maintained in the cells. The authors highlight the importance of YY1/TGFbeta signaling in the establishment of latency and conclude that this process is not possible in the absence of YY1 [135]. Furthermore, YY1 is involved in the regulation of herpesvirus reactivation through the modulation of cellular factors implicated in myeloid differentiation. One of these factors is the DEK protein. Sitwala et al. [136] showed that YY1 regulates the transcriptional activity of the DEK promoter by interacting with YY1-binding elements [136]. Moreover, the role of YY1 in transcriptional regulation was described in the varicella zoster virus (VZV). Khalil et al. [137] demonstrated that YY1 plays a role in the activation of IE62 viral protein-mediated gene expression. This cellular factor is involved in the activation of the gI, ORF10, and ORF28/29 viral promoters and plays a role in the lytic gene expression of VZV [137]. In addition, YY1 binds to the promoter elements of the Epstein–Barr virus and negatively controls the viral transcription process [72,94,95].

The role of YY1 in the life cycle of different viruses is presented in Table 2.

## 6. Concluding Remarks

The review summarizes the knowledge regarding the role of YY1 in viral life cycle and cancer. We particularly/notably emphasize the role of YY1 in the HPV life cycle and cervical cancer development. During HPV infection, YY1 regulates the expression of viral oncogenes *E6* and *E7* and is involved in the tumorigenesis process. Our review also shortly describes the oncogenic role of YY1 in different cancers such as head and neck squamous cell carcinoma and nasopharyngeal cancer, in which HPV infection is a well-known risk factor. Furthermore, we describe the dual role of YY1 (repressor or activator of gene transcription) in other viruses. To conclude, our work supports the current understanding of the role of the “dual face”, multifunctional YY1 protein in viral gene regulation.

## Figures and Tables

**Table 1:** YY1 expression in various HPV-related cancers.

Cancer	YY1 Expression Level	Mechanism	References
Oral cancer	Upregulated	Positive regulator of *CARM1* promoter	[103]
Esophageal squamous cell carcinoma (ESCC)	Upregulated	Coactivator to facilitate HO-1 transcription	[116]
Laryngeal cancer	Upregulated	Binding to MYCT1 promoter, inhibition of MYCT1 expression	[104]
Nasopharyngeal cancer (NPC)	Upregulated	Repressor of Trim26,inhibitor of miR-141 expression (mediated by c-Myc)	[105]

**Table 2:** Role of YY1 in the life cycle of different viruses.

Virus	YY1 Target	Function of YY1	Important Cellular or Viral Factors/Other Factors	References
Adenovirus (AAV)	P5 promoter	Binding to the P5 promoter and repression of viral transcription in the absence of E1A protein	-	[13]
Human immunodeficiency virus type 1 (HIV-1)	Long terminal repeat (LTR)	Repression of LTR expression and establishment of viral latency	LSF and histone deacetylase (HDAC)	[121]
	U3, U3R, and LTR	Transcriptional activator	HIV-1 Tat	[120]
Epstein–Barr virus (EBV)	BZLF1 (Zp) promoter	Repression of the BZLF1 (Zp) promoter and maintenance of EBV latency	-	[95,138]
Human cytomegalovirus (HCMV)	Major immediate early promoter (MIEP)	Binding to MIEP and repression of its activity during viral latency	Histone deacetylase (HDAC)	[134,135]
	*dek* promoter	Prevent myeloid differentiation that is crucial for viral reactivation	Nuclear Factor-Y (NF-Y)	[135,136]
Varicella zoster virus (VZV)	gI, ORF10, and ORF28/29 promoters	Binding to the YY1 sites and activation of IE62-dependent gene expression	Viral IE62 protein, cellular factors Sp1 and USF	[137]
Bovine coronavirus (BCoV)	*TLR7, TLR9, CEBPD, CEBPD, IRF1, IL-6,* and *RHOA* genes	Regulation of gene expression	-	[126]
Hepatitis B virus (HBV)	DGCR8 promoter	Downregulating the expression of DGCR8	Histone deacetylase (HDAC)	[129]
	19p13.11 enhancer	Regulating the transcriptional activity of HBV minichromosome	HBx	[131]
Human T lymphotropic virus type 1 (HTLV-1)	HTLV-1 R region	Binding to R sequence and leading to activation of gene expression	-	[119]
Moloney murine leukemia virus (M-MLV)	Trim28, negative control region (NCR)	Interaction with Trim28 protein, recruitment of Trim28 to NCR and mediation of the silencing of M-MLV	Trim28 protein	[124,139]
